# Genome-Wide Patterns of Adaptation to Temperate Environments Associated with Transposable Elements in Drosophila

**DOI:** 10.1371/journal.pgen.1000905

**Published:** 2010-04-08

**Authors:** Josefa González, Talia L. Karasov, Philipp W. Messer, Dmitri A. Petrov

**Affiliations:** Department of Biology, Stanford University, Stanford, California, United States of America; Fred Hutchinson Cancer Research Center, United States of America

## Abstract

Investigating spatial patterns of loci under selection can give insight into how populations evolved in response to selective pressures and can provide monitoring tools for detecting the impact of environmental changes on populations. Drosophila is a particularly good model to study adaptation to environmental heterogeneity since it is a tropical species that originated in sub-Saharan Africa and has only recently colonized the rest of the world. There is strong evidence for the adaptive role of Transposable Elements (TEs) in the evolution of Drosophila, and TEs might play an important role specifically in adaptation to temperate climates. In this work, we analyzed the frequency of a set of putatively adaptive and putatively neutral TEs in populations with contrasting climates that were collected near the endpoints of two known latitudinal clines in Australia and North America. The contrasting results obtained for putatively adaptive and putatively neutral TEs and the consistency of the patterns between continents strongly suggest that putatively adaptive TEs are involved in adaptation to temperate climates. We integrated information on population behavior, possible environmental selective agents, and both molecular and functional information of the TEs and their nearby genes to infer the plausible phenotypic consequences of these insertions. We conclude that adaptation to temperate environments is widespread in Drosophila and that TEs play a significant role in this adaptation. It is remarkable that such a diverse set of TEs located next to a diverse set of genes are consistently adaptive to temperate climate-related factors. We argue that reverse population genomic analyses, as the one described in this work, are necessary to arrive at a comprehensive picture of adaptation.

## Author Summary

The potential of geographic studies of genetic variation for the understanding of adaptation has been recognized for some time. In Drosophila, most of the available studies are based on *a priori* candidates giving a biased picture of the genes and traits under spatially varying selection. In this work, we performed a genome-wide scan of adaptations to temperate climates associated with Transposable Element (TE) insertions. We integrated the available information of the identified TEs and their nearby genes to provide plausible hypotheses about the phenotypic consequences of these insertions. Considering the diversity of these TEs and the variety of genes into which they are inserted, it is surprising that their adaptive effects are consistently related to temperate climate-related factors. The TEs identified in this work add substantially to the markers available to monitor the impact of climate change on populations.

## Introduction

The availability of genome sequences for an increasing number of organisms makes it possible to search for evidence of positive selection on an unprecedented scale. Several studies on different organisms such as bacteria, fruit flies, maize and humans suggest that positive selection is an important force shaping the genome [1]–[5]. However, how different forms of positive selection affect genome evolution and variation is still unclear. Particularly, we do not know the importance of directional selection, which promotes fixations of advantageous alleles, compared to that of spatially varying selection, which promotes maintenance of functional polymorphisms in populations. While most studies focused on the signatures of directional selection, new insights suggest that spatially varying selection might also be an important force [6]–[8]. Understanding the mechanisms and dynamics of spatially varying selection is important both from basic and applied perspectives since adaptive polymorphisms can be used as monitoring tools to detect the impact of climate change on populations [9]–[11].

Clines have long been used to infer the action of natural selection on particular genes and traits across environmental gradients [12]. *Drosophila melanogaster* is a good model to study adaptation in general and to environmental heterogeneity in particular because it is a tropical species that originated in sub-Saharan Africa and has only recently colonized the rest of the world [13]–[14]. Some of the adaptations that occurred in the populations that migrated out of Africa may specifically be related to temperate environments [15]–[18]. In this species, primarily populations collected along the Australian and North American East coast have been used to investigate the genetic variation associated with climatic adaptation [19]–[20]. Both geographical regions have proven to be ideal settings for this type of study because they span populations from tropical to temperate environments and flies can be easily collected at low altitudes thus avoiding the confounding effects of altitude on climate-associated patterns. Moreover, there are several lines of evidence suggesting that gene flow among populations along each one of these clines is high [19], [21]–[22]. This evidence favors the interpretation of significant genetic differentiation as a result of natural selection rather than being a by-product of non-adaptive processes related to population structure and history [23].

Adaptation to temperate environments in *D. melanogaster* has been related to a variety of genes [23]–[28], life-history traits [19],[29], stress resistance [30]–[31], thermotolerance [32] and morphological traits [20], [33]–[35]. However, most of these studies are based on *a priori* candidates, giving a biased picture of the genes and traits involved in adaptation to temperate environments. Studies that analyzed clines in allele frequencies also often lack an understanding of the selective agent responsible for producing the cline while in many cases the genes underlying the clines in phenotypic traits are unknown. Genome-wide analyses that not only identify candidate loci but also investigate the plausible selective agents and their phenotypic consequences are necessary in order to obtain a more comprehensive picture of adaptation to temperate environments.

In this study, we investigate adaptations to temperate environments in *D. melanogaster* associated with a specific class of mutation, the insertion of transposable elements (TEs). Potentially adaptive TEs are assessed on a genome-wide scale and based solely on their population behavior. There is strong evidence for the adaptive role of TEs in the evolution of the Drosophila genome [18], [36]–[38] and preliminary results based on the analysis of two populations collected at the endpoints of the Australian cline suggest that TEs may indeed play an important role in adaptation to temperate climates [18].

Our analysis is based on a genome-wide screen for TEs likely to be adaptive to the out-of-Africa environments. We first identified TEs likely to have increased in frequency during or after the spread of *D. melanogaster* out of Africa and therefore likely to be involved in adaptation to temperate climate. However, not all the identified TEs are equally likely to be adaptive. We used information about their family identity and about the patterns of nucleotide variability in the regions flanking these insertions to classify them in two groups: putatively adaptive and putatively neutral TEs. We then looked for evidence of population differentiation for both sets of TEs in three pairs of populations with contrasting climates. By themselves, such patterns may simply reflect genetic structure along the environmental gradients [19],[23]. However, while drift or historical processes predict similar population patterns for the neutral and adaptive TEs, selection predicts population differentiation patterns only for the adaptive TEs [23]. Therefore, we used the contrast between the two sets of TEs as evidence for the action of natural selection. We further evaluated the inference of the action of natural selection by testing for the consistency of the patterns on different continents. Once we identified the most likely TE candidates, we analyzed the association between their population frequencies and a number of climatic variables to gain insight into the environmental factors that might be contributing to selection. We ended our analysis by integrating all the information available for these TEs and their nearby genes to infer plausible phenotypic consequences of these mutations and the underlying mechanisms. We conclude that adaptation to temperate environments is widespread in Drosophila with TEs playing a significant role in this adaptation. We argue that without population genetics data of the kind described in this paper it is not possible to predict that such a diverse set of TEs located in such a diverse set of genes would be adaptive to temperate climate-related environmental variables. We believe that reverse population genomics studies as the one described in this work are necessary for a comprehensive understanding of adaptation.

## Results

### Genome-wide screen for TEs likely to be involved in adaptation during or after the spread of *D. melanogaster* out-of-Africa

We started our search from a set of 763 TEs annotated in the Release 5 of the *D. melanogaster* genome (Petrov, D.A., Fiston-Lavier, A.-S., Lipatov, M., Lenkov, K. and González, J., unpublished data). We identified TEs present at low frequencies in the ancestral African population and at high frequencies in the derived North American populations (see Materials and Methods). These TEs are likely to be involved in adaptation during or after the spread of *D. melanogaster* out of Africa. We further focused on TEs present in regions with a recombination rate larger than zero [39] as they are less likely to have reached high frequencies neutrally compared with regions of very low recombination where the efficacy of selection is substantially reduced [40]–[43].

Thirty-two TEs are likely to have increased in frequency during or after the migration of *D. melanogaster* out of Africa (Table S1). However, these 32 TEs are not equally likely to be adaptive. Some TEs belong to families in which the majority of TEs are present at high population frequencies. These families are more likely to be subject to relaxed purifying selection as a whole and therefore TEs in these families are more likely to have increased in frequency neutrally [42]. On the other hand, families in which the majority of TEs are present at low frequencies are likely to be subject to purifying selection. The few TEs present at high frequencies in these families are therefore likely to be adaptive. We used a maximum likelihood approach to estimate the selection coefficient of the 14 families represented in our dataset (see Materials and Methods; Table S2). We classified the families as putatively neutral when the estimated selection coefficients were not significantly different from zero and putatively adaptive when selection coefficients were significantly negative. TEs from families for which the selection coefficient could not be inferred due to the low number of elements in each family were also considered as possibly adaptive since we do not have clear evidence of their neutrality (Table S2).

The above classification into adaptive and neutral families is strongly supported by previous analyses [18],[44]. González et al. 2008 [18] analyzed the flanking regions of five putatively adaptive elements, including elements that belong to families with negative selection coefficients and elements from families for which selection coefficients could not be estimated. All five flanking regions showed evidence of selective sweeps suggesting that these TEs had increased in frequency due to positive selection [18],[44]. Four elements belonging to neutral families were also sequenced and all four appeared to have increased in frequency neutrally [44].

### Population patterns consistent with adaptation to temperate environments for adaptive, but not for neutral, TEs on different continents

**Figure 1:**
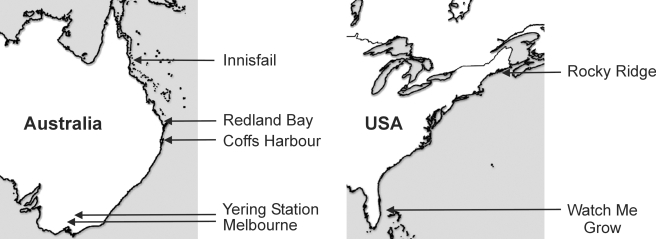
Geographical origin of the different *D. melanogaster* populations analyzed in this study.

Because *D. melanogaster* is a tropical species by origin [13]–[14], some of the adaptations that happened in the populations that migrated out of Africa may be related to adaptation to temperate environments [15]–[18]. If some of the TEs in our dataset are involved in adaptation to temperate habitats, we expect them to be present at higher frequencies in the out-of-Africa populations located in the more temperate regions compared to the ones located in the more tropical regions. Preliminary results support this hypothesis. We previously analyzed the frequency of 21 of these 32 TEs in populations collected in 2007 close to the endpoints of a latitudinal cline on the East coast of Australia. We found that eight of them were present at higher frequencies in the temperate compared to the tropical population [18]. To further test this hypothesis we estimated the frequency of the 32 TEs in populations collected close to the ends of latitudinal clines in Australia in 2007 (Innisfail and Yering Station) and in 2008 (Innisfail and Melbourne) and on the East coast of North America (Rocky Ridge and Watch Me Grow; Figure 1; see Materials and Methods). We expect TEs involved in adaptation to temperate environments to be present at higher frequencies in the Southern compared to the Northern populations in Australia while we expect the opposite pattern in North America: higher frequency in the Northern compared to the Southern populations. On the other hand, we do not have *a priori* reasons to expect directionality in the increase in frequency of putatively neutral TEs if population differentiation is present for these TEs.

We looked for evidence of population differentiation using a maximum likelihood approach (see Materials and Methods). However, some of the TEs that showed population differentiation are located inside one of the four cosmopolitan chromosomal inversions previously reported to show latitudinal patterns (Table S1) [45]. To avoid the confounding effects of the inversions on the population frequencies of these TEs, we scored by PCR the presence of three of the four inversions in all the strains analyzed (see Materials and Methods). Only a few strains showed presence of inversions *In(3L)Payne* and *In(2L)t* and these strains were removed from the analysis. Inversion *In(3R)Payne* showed a strong clinal pattern as previously described [46]–[47]. Because the confounding effects of this inversion could not be discarded, TEs located inside inversion *In(3R)Payne* (FBti0019415, FBti0019410 and FBti0019418) and inside inversion *In(2R)NS* (FBti0019012), which was not scored, were excluded from our analysis.

**Figure 2:**
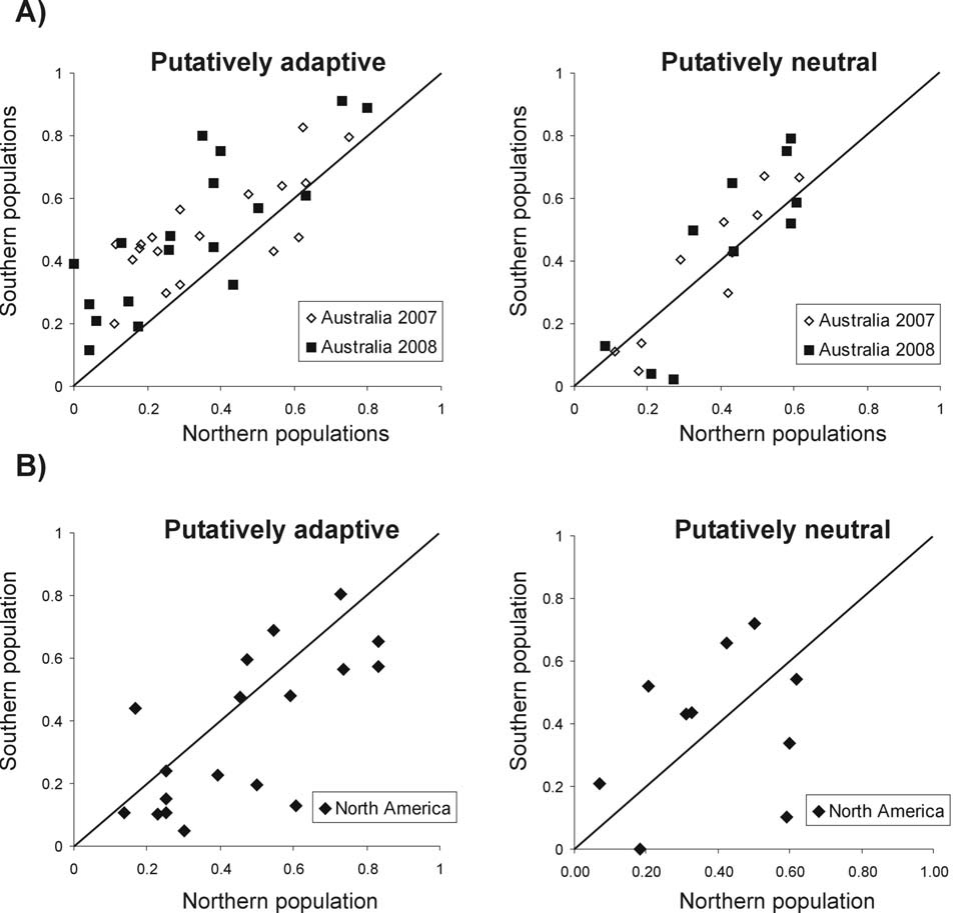
Frequencies of the putatively adaptive and putatively neutral TEs. In the northern versus the southern populations in Australia (A) and North America (B).

Table 1 shows the frequency of the 28 TEs for which we could discard the confounding effects of inversions in the three pairs of populations analyzed. The first 18 TEs in the list belong to putatively adaptive families and the last 10 belong to putatively neutral families. We plotted the frequency of each of the 28 TEs in the Northern *vs* the Southern populations for the Australian (Figure 2A) and the North American data (Figure 2B) both for putatively adaptive and for putatively neutral elements. Adaptive TEs are present at higher frequencies in the Southern compared to the Northern Australian populations (G-test, *P-value* = 0.0001) while neutral TEs are not (G-test, *P-value* = 0.3). The same pattern was found when the populations collected in different years were considered independently (G-test, *P-value* = 0.045 for adaptive TEs for both years and *P-value* = 0.3 and 0.4 for neutral TEs in 2007 and 2008 respectively). The difference between putatively adaptive and putatively neutral TEs is significant (G-test, *P-value* = 0.0072).

**Table 1:** Population frequencies of the 28 TEs analyzed in this study.

			Australia 2007				Australia 2008				North America			
					Raw	FDR^b^			Raw	FDR			Raw	FDR
Flybase ID	Family	Chr^a^	North	South	P-value	P-value	North	South	P-value	P-value	North	South	P-value	P-value
FBti0018880	Bari1	2R(55F8)	0.48	0.61	0.2017	0.4344	0.50	0.57	0.5262	0.6405	0.55	0.69	0.1767	0.2748
FBti0019056	pogo	X(12F1)	0.61	0.48	0.2000	0.5090	0.38	0.65	**0.0098**	**0.0458**	0.47	0.60	0.2757	0.3509
FBti0019065	pogo	X(13C1)	0.21	0.48	**0.0108**	0.0758	0.43	0.33	0.2823	0.4392	0.73	0.80	0.3870	0.4515
FBti0019144	Rt1b	2L(31A3)	0.18	0.44	**0.0449**	0.1573	0.06	0.21	0.0676	0.1578	0.17	0.44	**0.0113**	**0.0395**
FBti0019164	X-element	2L(34A5)	0.34	0.48	0.2011	0.4693	0.35	0.80	**6e-05**	**0.0008**	0.39	0.23	0.0911	0.1594
FBti0019170	F-element	2L(34C4)	0.18	0.45	**0.0063**	0.0885	0.26	0.43	0.1240	0.2170	0.25	0.24	0.9025	0.9025
FBti0019372	S-element	3R(88A4)	0.16	0.40	**0.0103**	0.0959	0.04	0.26	**0.0024**	**0.0168**	0.25	0.11	0.0770	0.1539
FBti0019386	invader4	3R(89B7)	0.23	0.43	**0.0400**	0.1867	0.13	0.46	**0.0004**	**0.0042**	0.50	0.20	**0.0017**	**0.0080**
FBti0019430	Doc	3R(96D1)	0.75	0.80	0.6107	0.7772	0.73	0.91	**0.0205**	0.0819	0.74	0.57	0.0881	0.1645
FBti0019443	Rt1b	3R(98B3)	0.11	0.45	**0.0003**	**0.0077**	0.00	0.39	**6e-08**	**1.7e-06**	0.14	0.11	0.6666	0.7179
FBti0019624	hopper	X(10B2)	0.55	0.43	0.2858	0.5001	0.26	0.48	**0.0324**	0.1134	0.45	0.48	0.8308	0.8615
FBti0019627	pogo	X(10C6–7)	0.57	0.64	0.5201	0.7665	0.63	0.61	0.8767	0.9092	0.83	0.57	**0.0109**	**0.0435**
FBti0019679	1731	X(20A1)	0.63	0.65	0.7840	0.8443	0.38	0.44	0.5699	0.6649	0.59	0.48	0.2826	0.3441
FBti0019747	F-element	X(20E2)	0.25	0.3	0.5559	0.7782	0.17	0.19	0.8406	0.9053	0.23	0.10	0.1083	0.1783
FBti0020042	jockey	3L(64D3)	0.11	0.2	0.2075	0.4150	0.04	0.12	0.3256	0.4558	0.25	0.15	0.2736	0.3648
FBti0020046	Doc	3L(65A3)	0.29	0.33	0.7339	0.8220	0.15	0.27	0.1671	0.2752	0.30	0.05	**0.0016**	**0.0087**
FBti0020091	Rt1a	3L(68C1)	0.63	0.83	**0.0433**	0.1732	0.80	0.89	0.3165	0.4664	0.83	0.65	**0.0426**	0.1086
FBti0020119	S-element	3L(71E1)	0.29	0.56	**0.0203**	0.1137	0.40	0.75	**0.0037**	**0.0206**	0.61	0.13	**9.6e-07**	**1.3e-05**
FBti0018879	BS	2R(58E2)	0.61	0.67	0.6230	0.7585	0.43	0.65	**0.0356**	0.1107	0.60	0.34	**0.0168**	0.0523
FBti0019079	BS	X(17A2)	0.18	0.14	0.5594	0.7459	0.08	0.13	0.4934	0.6579	0.20	0.52	**0.0015**	**0.0108**
FBti0019133	BS	2L(28B2)	0.50	0.55	0.6584	0.7682	0.59	0.79	0.0740	0.1594	0.62	0.54	0.4579	0.5128
FBti0019165	BS	2L(34B1)	0.41	0.52	0.2859	0.4708	0.59	0.52	0.5196	0.6612	0.43	0.66	**0.0382**	0.1069
FBti0019604	BS	X(7E4)	0.43	0.43	0.9757	0.9757	0.43	0.43	0.9774	0.9774	0.50	0.72	0.0579	0.1246
FBti0019771	1360	2L(36C6)	0.42	0.3	0.2563	0.4784	0.33	0.50	0.0893	0.1787	0.59	0.10	**3.5e-07**	**9.7e-06**
FBti0020056	BS	3L(65D6)	0.18	0.05	0.0695	0.2163	0.27	0.02	**0.0364**	0.1020	0.07	0.21	**0.0478**	0.1116
FBti0020057	BS	3L(65E4)	0.29	0.41	0.3054	0.4751	0.58	0.75	0.0999	0.1865	0.31	0.43	0.2398	0.3534
FBti0020125	BS	3L(73A4)	0.11	0.11	0.9406	0.9754	0.21	0.04	0.0560	0.1426	0.18	0.00	**0.0010**	**0.0095**
FBti0020155	1360	3L(75E1-2)	0.52	0.67	0.1357	0.3801	0.61	0.59	0.8316	0.9314	0.33	0.44	0.2658	0.3721

The horizontal line separates TEs that belong to putatively adaptive families (top 18 TEs) from TEs that belong to putatively neutral families (bottom 10 TEs).

**a** Chromosome location.

**b** False Discovery Rate.

As predicted, in North America putatively adaptive TEs tend to be more frequent in the Northern compared to the Southern populations although this pattern is marginally nonsignificant (G-test, *P-value* = 0.0845). However, compared to neutral TEs, adaptive TEs are more frequent in the Northern population (G-test, *P-value* = 0.0235). The observed patterns are consistent between continents (G-test, *P-value* = 0.066 and 0.17 for adaptive and neutral TEs respectively).

In summary, the contrasting results between putatively adaptive and putatively neutral TEs and the consistency of patterns between continents strongly suggest that putatively adaptive TEs play a role in adaptation to temperate environments.

### Identifying the most likely TE candidates to be involved in adaptation to temperate environments

The majority of putatively adaptive TEs show population differentiation patterns consistent with adaptation to temperate climates when considered one by one in at least one of the three pairs of populations analyzed (Table 1). It is possible that most of these TEs are involved in adaptation to temperate environments: the different levels of significance could simply reflect the differences in the selective advantage these TEs confer to the organism. However, because we are interested in identifying the strongest candidates for further analysis, we focused on those TEs that are significant after correction for multiple testing [48].

There are 12 TEs that show significant patterns of population differentiation after correction for multiple testing, ten of which show the pattern expected if they are involved in adaptation to temperate environments (Table 1). Nine of these 12 TEs are putatively adaptive (Table 1) and eight of them show a pattern consistent with adaptation to temperate environments. Furthermore, FBti0019386 and FBti0020119 show consistent population differentiation patterns in the two hemispheres (Table 1). As mentioned above, replicate observations of differentiation on two continents are considered to be strong evidence for selection [25],[49]. The absence of replicate observations, however, does not preclude selection (see Discussion).

Although when considered together neutral TEs were not present at higher frequencies in temperate compared to tropical populations (Figure 2), there were three individual TEs that showed significant population differentiation patterns after correction for multiple testing. In all three cases the population differentiation patterns were only found in the North American populations and only two of the three showed patterns consistent with adaptation to temperate environments (Table 1).

Finally, putatively adaptive TEs that did not show patterns of population differentiation could be adaptive to conditions that are common to both temperate and tropical out-of-Africa populations (Table 1). For example, FBti0019430 has been shown to confer resistance to pesticides [37] although a recent analysis suggests that this was not the selective reason for its spread (Aminetzach Y. T., Karasov T.L. and Petrov D. A., unpublished data).

### Environmental factors likely to contribute to adaptation to temperate climates

**Figure 3:**
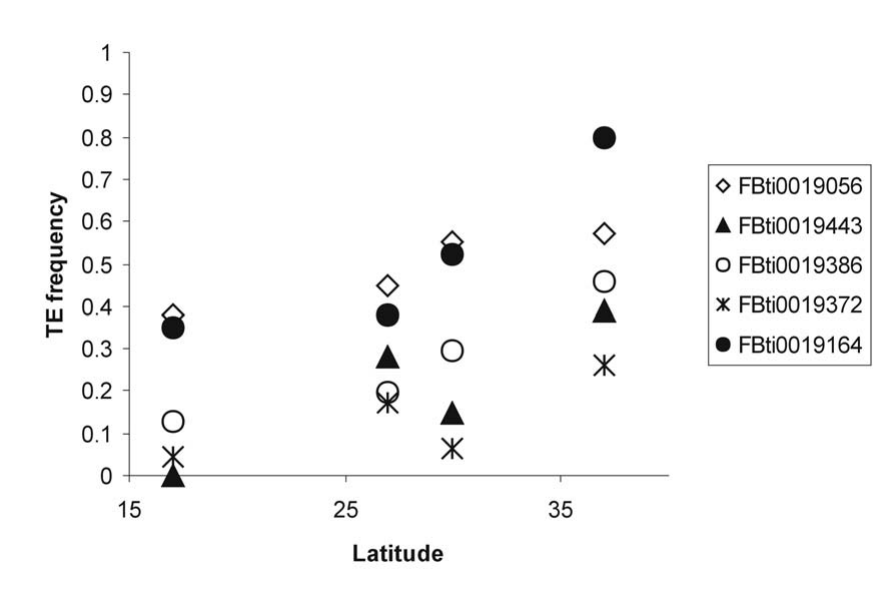
Frequency of five TEs that showed significant patterns of population differentiation in four populations collected along the east coast of Australia in 2008.

We performed additional latitudinal and climatic analysis to investigate potential environmental agents that might be contributing to selection. These analyses were restricted to the six TEs that showed significant population differentiation patterns in the Australian 2008 populations. In order to establish clinal patterns, it is important to analyze flies that have been collected over a narrow period of time because clines can vary seasonally [50]. We only have such populations for the 2008 Australian cline. The location of the four Australian populations analyzed, Innisfail, Redland Bay, Coffs Harbour and Melbourne, is shown in Figure 1. FBti0020119 was not included in the final analysis since we could not discard the confounding effect of inversion *In(3L)P* on its frequency (Figure 3 and Table S3).

We used regression analysis to test the association between population frequencies and latitude (see Materials and Methods). We also analyzed the association between population frequencies and climatic variables that have previously been shown to be related to geographic variation [24]–[28]: mean maximum temperature (Tmax), mean minimum temperature (Tmin) and mean rainfall (see Materials and Methods). Three TEs show associations with latitude, Tmax or both: FBti0019386 shows significant association with latitude and Tmax, FBti0019056 shows a marginally non-significant association with latitude and a marginally significant association with Tmax and FBti0019164 shows a marginally non-significant association with Tmax (Table 2). FBti0019443 shows a significant association with both Tmin and rainfall (Table 2). Considering a 5% false discovery rate level, we would expect only one of the 20 tests performed to be significant. However, we obtained five significant tests suggesting that the majority of these tests are indeed significant.

**Table 2:** Regression analysis examining associations between five TEs sampled in four Australian populations and different environmental variables.

Flybase ID	Frequency versus latitude		Frequency versus mean Tmax		Frequency versus mean Tmin		Frequency versus mean rainfall	
	R^2^	P-value	R^2^	P-value	R^2^	P-value	R^2^	P-value
FBti0019056	0.8869	0.058	**0.9046**	**0.049**	0.8162	0.096	0.8178	0.096
FBti0019443	0.8483	0.078	0.6991	>0.05	**0.9431**	**0.028**	**0.9373**	**0.032**
FBti0019386	**0.9178**	**0.0420**	**0.9893**	**0.0053**	0.7894	>0.05	0.8163	>0.05
FBti0019164	0.4807	>0.05	0.8980	0.052	0.6115	>0.05	0.6496	>0.05
FBti0019372	0.6380	>0.05	0.5486	>0.05	0.6753	>0.05	0.6961	>0.05

Overall, we found that four of the five TEs analyzed show weak clinal patterns. We believe that the weakness of these patterns is at least partly due to a lack of power given the small number of populations analyzed. Our results are therefore suggestive of clinal patterns but they are not conclusive. More populations need to be analyzed in order to get a better insight into which factors are relevant and to analyze the possible interactions among those factors.

The change in TE population frequency associated with temperature may be reflecting temperature-dependent selection. However, this association could also be due to the selective action of other ecological variables that correlate with temperature such as food resources, the presence of competitors, predators, or pathogens or other unknown variables. In the case of association of population frequencies with rainfall, it is difficult to explain it in terms of direct selective effects of this climatic variable. Many indirect effects are however possible because rainfall influences many aspects of the physical, chemical and biological environment [25].

### Genes and traits under selection

To learn more about the genes and traits likely to be under selection, we analyzed the available functional information for the neighboring genes of the 10 most likely TE candidates (Table 3). We used Fatigo to look for Gene Ontology terms under- or over-represented in this set of genes compared to the rest of genes in the genome [51]. We did not find significant under- or over-represented functional terms, which may be explained by the small number of genes together with the sparse functional annotation available for Drosophila genes.

**Table 3:** Characteristics of the 10 TEs associated with adaptation to the temperate environments.

Flybase ID	Class	Family	Size	Comp. to canonical TE^a^	Chr^b^	Pop. diff.^c^	Location to closest gene (R5.19)	GO annotations		Other functional information
								Biological process	Molecular function	
FBti0019056	TIR	pogo	185	internal deletion	X	AU2008	3rd intron of CG9413	amino acid transport	amino acid transmembrane transporter	Immune response [83]
FBti0019164	LINE-like	X-element	180	5′ truncated	2L	AU2008	2nd intron of *CG9932*	proteolysis	metallopeptidase	Associated with starvation resistance and locomotor reactivity [84]
FBti0019372	TIR	S-element	1761	full length	3R	AU2008	1st intron of *rdx*	segment polarity determination/eye development/regulation of proteolysis	protein binding	Mitotic cell cycle defective [85]
FBti0019386	LTR	invader4	346	solo LTR	3R	AU&NA	1st intron of *sra*	regulation female receptivity/egg activation/olfactory learning	protein binding	Meiotic cell cycle defective [86]/Courtship defective [66]
FBti0019443	LINE-like	Rt1b	3074	5′ truncated	3R	AU2007-08	3rd intron of *CG34353*	none	none	Circadian regulated gene [67]
FBti0019627	TIR	pogo	185	internal deletion	X	NA	3′ UTR of *Kmn1*	Chromosome segregation	none	Mitotic cell cycle defective [87]
FBti0019771	TIR	1360	1105	internal deletion	2L	NA	23.2 kb 5′ of *CG34170*	none	none	none
							26.8 kb 5′ of *CG31804*	none	none	Specifically regulated by HP1 in males [88]
FBti0020046	LINE-like	Doc	2304	5′ truncated	3L	NA	268bp 3′ of *Jon65Aiv*	proteolysis	S-type endopeptidase	Odor-guided behaviour [69] Mating-regulated gene [89] Immune responsive gene [90]
							8.1 kb 5′ of *Jon65Aiii*	proteolysis	S-type endopeptidase	Odor-guided behaviour [69]
FBti0020119	TIR	S-element	1731	full length	3L	AU&NA	1st intron of *Ago2*	defense response to virus/RNA interference	protein binding	none
FBti0020125	LINE-like	BS	5123	full length	3L	NA	411bp 5′ of *CG42513*	none	adenylate cyclase	none
							1.5 kb 3′ of *CG12436*	none	none	

**a** Comparison to canonical TE.

**b** Chromosome location.

**c** Population differentiation: Australia (AU), North America (NA).

Some of the identified TEs are inserted into or close to genes involved in processes that have previously been shown to be under positive selection such as metabolism, defense response, or cell cycle [7],[20],[52]. This gives us confidence that our procedure is identifying promising cases, since these TEs were identified based exclusively on their population behavior without taking into account the description of their neighboring genes.

### Characteristics of the TEs involved in adaptation to temperate climates: how these TEs might be affecting the expression of their nearby genes

To try to understand how TEs might affect the expression of their nearby genes, we analyzed several molecular and functional characteristics of the insertions. We restricted this analysis to the eight putatively adaptive TEs as they are likely to be the causative mutations. These eight TEs are distributed across all three major chromosomes and are unlinked with each other suggesting that they constitute independent cases of adaptive differentiation between temperate and tropical populations (Table 3). The three main classes of TEs, LTR, LINE-like and TIR, are represented in this dataset [53].

Six of the TEs are located in introns, one in a UTR and one in an intergenic region suggesting that they are involved in regulatory changes (Table 3). Indeed, for three of these TEs there is experimental evidence suggesting that they are affecting the expression of nearby genes (FBti0019372, FBti0020019 and FBti0020046) [18]. We compared the regions where they are inserted between *D.melanogaster* and *D. simulans*. We used the VISTA browser default parameters to examine the pairwise alignment of these regions [54]. For two of them, FBti0020046 and FBti0020119, the sequence conservation drops in the region immediately adjacent to the insertion. This suggests that rather than disrupting existing regulatory elements these TEs might affect expression by adding regulatory elements themselves. The other six TEs are inserted in conserved regions suggesting that they might disrupt existing regulatory elements and/or add regulatory elements.

Other than the mechanisms mentioned above, TEs inserted into introns may be affecting gene expression by driving antisense transcription, by interfering with normal splicing patterns of the mRNA or by being incorporated as exons. We tested the latter mechanism by searching for chimeric gene-TE ESTs using the modENCODE genome browser available at http://flybase.org. We found several ESTs containing these TEs but none of them contained genic sequences as well.

Only one TE, FBti0019627 is inserted into the 3′ UTR of a gene, *Kmn1*. 3′ UTRs regulate several aspects of gene expression such as mRNA decay and the spatial and temporal patterns of expression [55]–[57]. There are several ways by which a TE inserted in a 3′UTR can affect gene expression. We first looked for the presence of alternative poly(A) signals in the sequence of FBti0019627 and we did not find any. However, we identified a U-rich sequence in this TE that could be acting as a downstream element (DSE). Since FBti0019627 is inserted 42 bp downstream of the poly(A) signal, the presence of a DSE in its sequence could be altering the place where the cleavage of the mRNA takes place [58]. We found an EST that is consistent with the use of this new DSE: it contained only a fragment of the TE (113 bp) and it did not include the DSE. We also found an EST containing the whole TE (186 bp) and an EST that ends 3 bp after the poly(A) signal. These results suggest that flies with the insertion produce three mRNAs that differ in the length of the 3′ UTR and therefore potentially differ for example in binding sites for miRNA or for RNA-binding proteins [55],[59].

Another possibility is that these eight TEs affect gene expression by co-mobilizing DNA when they transposed. There is evidence for co-mobilization of DNA for non-LTR elements [60]–[62] and for transposons [63]. DNA mobilization by non-LTR elements is normally due to read-through transcripts that lead to co-mobilization of their 3′ flanking DNA [60]. However, mobilization of DNA in the 5′ end of the element has also been described [62]. We analyzed the TE sequences and their flanking regions and found no evidence for co-mobilization of DNA (Table 3). Two of the three LINE-like elements are flanked by target site duplications (TSDs) and the sequence between the TSDs only show homology to other TEs in the same family. Although the other LINE-like element, FBti0020046, is apparently not flanked by TSDs, the analysis of its flanking regions revealed that the 14 bp 5′ to the annotated TE show homology with a INE-1 element. These 14 bp are followed by a TSD. Again the region between the TSDs only shows homology with other TEs in the same family. The four TIR elements are either full length or show internal deletions compared to their canonical element, and the only LTR element is a solo LTR suggesting that none of these TEs co-mobilized DNA.

Further analyses such as looking for evidence of antisense transcription driven by these TEs or the existence of different splicing variants in flies with and without the insertions are needed in order to elucidate the mechanisms by which these TEs may be affecting gene expression.

## Discussion

### Adaptation to temperate environments is widespread in Drosophila

In this work, we identified a set of TEs that are likely to be involved in adaptation during or after the spread of *D. melanogaster* out of Africa (Table 1). Because this species is tropical by origin, some of these adaptations may specifically be related to adaptation to temperate climates [15]–[18]. To test this prediction, we estimated the population frequencies of these TEs in populations with contrasting climates that were collected near the endpoints of two known latitudinal clines in Australia and North America (Figure 1). If some of these TEs are involved in adaptation to temperate climates we expect them to be present at higher frequencies in temperate compared to tropical populations. However, other than being caused by selection, patterns of population differentiation may simply be the by-product of non-adaptive processes related to population structure and history [19],[23]. To distinguish between these two possibilities, we looked for patterns of population differentiation not only in the set of TEs identified as putatively adaptive but also in a comparable set of neutral TEs. While drift, isolation by distance or historical processes should affect the patterns of variation across the entire genome and therefore affect both neutral and adaptive TEs, we expect selection to affect only the adaptive loci.

As mentioned above, the two sets of TEs, adaptive and neutral, are comparable: they are all present at low frequencies or absent in AF populations and are present in all the NA populations analyzed (Table S1). However, neutral TEs come from families with selection coefficients not significantly different from zero while adaptive TEs belong to families with selection coefficients significantly negative or from families for which we do not have a clear evidence of neutrality (Table S2). In addition, while patterns of polymorphism around the adaptive TEs show signatures of selection, the regions flanking neutral TEs suggest that these TEs have increased in frequency neutrally [18],[44]. As predicted if selection is the cause of population differentiation patterns, TEs classified as putatively adaptive are present at higher frequencies in temperate compared to tropical populations while putatively neutral TEs are not (Figure 2). Furthermore, these patterns of population differentiation are consistent between years and between continents (Figure 2). If population differentiation patterns were entirely random, they would be unlikely to occur in both hemispheres [25],[49]. Therefore, the contrasting results for adaptive and neutral TEs and the repeatability of population differentiation patterns across continents strongly suggest that selection is responsible for the observed population patterns.

We used a maximum likelihood approach to identify the most likely TE candidates to be involved in adaptation to temperate environments. After correcting for multiple testing, we found 12 TEs with significant patterns of population differentiation. Ten of them were present at higher frequencies in temperate populations as expected if they are involved in adaptation to temperate environments (Table 3). Two of these 10 TEs show parallel patterns on the two continents. However, the absence of replicate observations for the other TEs does not preclude selection. It could simply reflect a lack of statistical power due to the limited number of strains analyzed. The difference between continents may also be due to differences in the climatic gradients. Indeed, the latitudinal range spanned by Australian populations is 17° to 37° while North American populations range from 27° to 44°. The absence of parallel clines on the two continents could also be related to differences in the genetic background of Australian and North American populations. These two continents had very different histories of colonization: *D. melanogaster* spread into North America in the past few centuries while it spread into Australia only in the last 100 years [13],[64]. Finally, another possibility is that these differences are due at least in part to different patterns of isolation by distance in the two continents. However, there are several lines of evidence that suggest that gene flow among populations along each one of these clines is high [19], [21]–[22].

Our results, based on the analysis of the TEs annotated in the sequenced *D. melanogaster* strain, suggest that adaptation to temperate environments is widespread in Drosophila. Although this strain has been described as a “typical” *D. melanogaster* strain [53], it would be interesting to analyze the population dynamics of TEs annotated in other strains. The sequencing of 192 *D. melanogaster* strains currently in progress will facilitate this analysis (http://www.hgsc.bcm.tmc.edu).

### Putatively adaptive TEs are likely to be the causative mutation

Putatively adaptive TEs are likely to be the actual causative adaptive mutations, and not just linked to a nearby adaptive mutation. If the population differentiation of a putatively adaptive TE were due to linkage to a nearby adaptive mutation, then the direction of the differentiation should only depend on whether the adaptive mutation emerged on a haplotype with the TE, or one without it. Both directions should therefore be possible. If, however, the TE is the adaptive mutation itself, then the TE's frequency is always expected to be higher in the more temperate population. Indeed, eight out of nine putatively adaptive TEs that showed significant population differentiation are present at higher frequencies in temperate populations (Table 1). On the other hand, among the three putatively neutral TEs two were more frequent in the temperate population and one was more frequent in the tropical population. Although the numbers are small, this pattern is consistent with the population differentiation of the neutral TEs being due to linkage to a causative mutation. Additional evidence comes from the previously mentioned studies by González et al. 2008 [18] and Macpherson et al. 2008 [44], where patterns of nucleotide variability around TEs were analyzed. In every investigated instance, the high frequency of putatively adaptive TEs was found to be consistent with positive selection while putatively neutral TEs were always confirmed to have increased in frequency neutrally.

It is also possible that some of the TEs in the neutral families are actually adaptive. To elucidate whether TEs in neutral families are linked to an adaptive mutation or adaptive themselves, and to completely discard the existence of linked adaptive mutations in the vicinity of adaptive TEs, the flanking regions of each one of these 10 TEs should be analyzed. The future availability of the whole genome sequences for 192 *D. melanogaster* strains should facilitate this analysis (http://www.hgsc.bcm.tmc.edu/). In any case, the genomic regions where TEs showing significant population differentiation are inserted represent strong candidate regions to be involved in adaptation to temperate climates and deserve further study. They also add significantly to the set of candidate loci already available to study and monitor the impact of climate change on populations [65].

### What are the phenotypic consequences of the adaptive insertions?

The adaptive TEs reported here span the range of TE diversity in *D. melanogaster* (Table 3). The putatively affected genes are also highly diverse in terms of their molecular and cellular functions (Table 3). It would be natural to assume that the resulting adaptive effects are diverse as well and have evolved in response to multiple unrelated selective pressures associated with the migration out of Africa. In contrast, our results suggest that the selective behavior of these adaptive TEs can be largely explained by latitude. These results might thus be revealing cryptic simplicity of the adaptive process in *D. melanogaster* – much of it might be about latitude – and challenge us to understand how diverse genes and processes can all generate adaptive effects in response to a related set of selective pressures.

Taking into account both the functional information of the genes located nearby (Table 3) and the information on the possible selective factors reported in this work (Table 2), we can construct plausible hypotheses about the phenotypic consequences of the adaptive insertions. For example FBti0019386, which shows population differentiation in both continents, is inserted into a conserved region in the first intron of *sra* (Table 3). Variation in the population frequency of this TE is associated with latitude and Tmax. Changes in the expression level of this gene critically affect ovulation and female courtship [66]. We therefore speculate that the insertion of FBti0019386 into *sra* might affect fecundity specifically at low temperatures.

FBti0019443 is inserted in a circadian-regulated gene, *CG34353* [67]. The population frequency of this TE is associated with Tmin and rainfall. Some authors have suggested that differences in latitude challenge the circadian clock because of the associated changes in temperature and photoperiod [68]. There is no information about the biological process or the molecular function of *CG34353*, but we suspect that temperature and photoperiod may play a role in its evolution. Components of fitness such as male and female fertility, survival rates throughout development or stress resistance should be analyzed under different temperatures and photoperiods in order to link this insertion to its phenotypic consequence.

Another example is FBti0020046, which is inserted in the intergenic region between *Jon65Aiv* and *Jon65Aiii*. Both genes have been associated with odor-guided behavior [69]. One important factor involved in the ability to colonize new habitats is the capacity of using different food resources [70]. The shift from a natural source to a domesticated fruit in the fly *Rhagoletis pomonella* is associated with, and perhaps causally related to, a shift in olfactory preferences [71]. We can therefore speculate that the insertion of this TE in the intergenic region of genes involved in olfactory-guided behaviour played a role in the ability of *D. melanogaster* to use different food resources. However, olfactory behavior is also involved in other processes such as avoidance of environmental toxins and predators, mate selection or reproduction [72]. Any of these processes could therefore have been affected by changes in the expression of these genes.

Pleiotropic effects of adaptive mutations, as the ones just described, can severely complicate the identification of the phenotypic trait on which selection is acting even when we have clues about the potential interesting phenotypes. This is exemplified by the analysis of the *Bari-Jheh* insertion previously carried out in our lab [38]. This TE is inserted between genes involved in Juvenile Hormone metabolism. Juvenile Hormone has major effects on various aspects of development and life history traits [73]. Although we were able to find subtle consequences of this insertion on life history traits that were consistent with the reduced expression of the nearby genes, we could not pinpoint which of the phenotypic effects of the insertion was adaptive. Another factor that can severely complicate the detection of selection in experimental populations is that the observed changes in TE frequency may be explained not by a single environmental variable but by a combination of them [74]–[75]. Finally, although having identified both the phenotypic trait and the relevant environmental conditions, the fitness differences between the flies with and without the insertion might be too small to be experimentally detected [76]. Because adaptive mutations might be difficult to study at the phenotypic level, reverse population genomics analyses as the one described in this work which allow detection of a consistent response of a set of adaptive mutations to the environment are necessary to obtain a comprehensive picture of adaptation.

### Conclusions

We found patterns of population differentiation associated with TE insertions that are consistent with the model of an ancestral African species adapting to temperate climates. Our results suggest that adaptation to temperate climates in Drosophila is widespread with TEs playing a significant role in this adaptation. Considering the variety of TEs in our set, it is remarkable that their adaptive effects seem to be consistently associated with climate-related selective pressures, potentially revealing cryptic simplicity of the adaptive process in *D. melanogaster*. We identified the most likely TE candidates and integrated information on population behavior, possible environmental selective agents and both molecular and functional information of the nearby genes to infer the plausible phenotypic consequences of these insertions in the environment in which they evolved. Our long term objective is to experimentally measure the phenotypic differences between flies with and without these insertions which will help us to understand their adaptive effects. Both reverse population genomic analyses of the kind described in this work and functional analysis that link the identified mutations to their adaptive phenotypes are necessary to arrive at a comprehensive picture of adaptation.

## Materials and Methods

### Dataset

In a previous work, we used the Release 3 annotation of TEs in the *D. melanogaster* genome to design primers to check the population frequency of individual TEs in the genome [18]. Using a pooled-PCR approach, we obtained frequency data for a total of 902 TEs in five North American populations (64 strains combined in six pools) and one sub-Saharan (Malawi) African population (11 strains combined in one pool; for details see González et al. (2008) [18]). Release 5 corrected the annotations for a large number of TEs relative to Release 3 and the PCR results obtained previously were updated accordingly (Petrov, D.A., Fiston-Lavier, A.-S., Lipatov, M., Lenkov, K. and González, J., unpublished data). In this work, we used the updated version of our database, containing information for 763 TEs, to re-run the query designed to search for TEs that may have contributed to adaptation during and/or after the migration of *D. melanogaster* out of Africa. Specifically, we looked for insertions that (1) were present in all six North American pools (199 TEs), (2) were not fixed in the African pool (85 TEs) and (3) were present in regions of the genome with a recombination rate larger than zero [39] (45 TEs, Table S1). Our results are not very sensitive to the exact value of these cutoffs. If we consider the TEs present in five North American pools instead of six pools, the number of TEs varies from 199 to 226. However, the estimated population frequency for TEs present in 5 pools is only 5.1%–35% [77]. Since these TEs might be at low population frequencies we decided to focus on those ones present in all six North American pools (estimated population frequency 11%–100%; [77]). Varying the recombination rate cutoff between 0 cM/Mb and 1 cM/Mb only changes the number of TEs from 45 to 42 (Table S1).

A total of 45 TEs matched the above criteria (Table S1). We then estimated the frequency of those TEs in the Malawi population using PCR with individual strains (see below; Table S4). We filtered out TEs present in ≥30% of the strains analyzed because those TEs are less likely to be involved in adaptation to the out-of-Africa environments and we ended with a dataset of 32 TEs (Table S1). Again, varying the cutoff value for example from 30% to 15% only changes the results marginally (from 32 TEs to 30 TEs; Table S1).

Although all the selected TEs were found to be present at low frequencies in the Malawi population, it is possible that they are present at high frequencies in other sub-Saharan African populations because only 11 strains were sampled and because there might be substantial substructure in the *D. melanogaster* population in sub-Saharan Africa [78]. In previous works in our laboratory, we extended the analysis of TEs found to be absent or present at low frequencies in Malawi to three other sub-Saharan populations, two from Zimbabwe and one from Kenya (Table S4; [18],[79]). We found that putatively adaptive TEs that were absent or present at low frequencies in Malawi were also absent or present at low frequencies in the three additional African populations analyzed (Table S5). Similar results were obtained for putatively neutral TEs (Table S5). Overall, the analyzed TEs seem to have increased in frequency either during or after the expansion out of Africa.

### Drosophila stocks

In addition to the African *D.melanogaster* stocks mentioned above, the following populations collected along the Australia and North America East coast clines were analyzed in this study (Figure 1; Table S4): two Australian populations collected in 2007 in Innisfail in North Queensland and Yering Station in South Victoria. Four Australian populations collected in 2008 in Innisfail in North Queensland, Redland Bay in Queensland, Coffs Harbour in New South Wales and Melbourne in South Victoria. Two North American populations collected in Rocky Ridge in Bowdoinham, Maine, USA, and Watch Me Grow in Ft. Pierce, Florida, USA.

### Checking for presence/absence of TEs

The presence and/or absence of the TEs analyzed in this work was determined using PCR. Two different sets of primers were used: one set was intended to assay for the presence of the TE and consisted of a “Left” primer which lay within the TE sequence and a “Right” primer that lay in the flanking region to the right of the insertion. We expect this PCR to give a band only when the element is present. The other set of primers was intended to assay for the absence of the element and consisted of a “Flank” primer which lay in the flanking region to the left of the element and the “Right” primer mentioned above. In this case, the absence of the TE should give a shorter “absence” band and the presence of the TE should give a longer “presence” band. We assumed that the “presence” band is unlikely to be amplified if the TE is longer than 800bp.

### Maximum likelihood estimation of TE population frequencies

Populations were sampled only a few generations after they were collected in the field. This is important since TE frequencies may change due to laboratory selection or laboratory bottlenecks and therefore laboratory frequencies might not be representative of the field frequencies [50]. Moreover, we did not find significant differences in the population frequencies of the analyzed TEs between the two years in the Northern or Southern Australian populations (Table S6). In any case, we expect the changes due to lab conditions to affect the Northern and Southern populations similarly (and the adaptive and neutral TEs similarly as well) since these populations were maintained under the same laboratory conditions.

We estimated the frequency of each one of the 32 TEs in each population using PCR with individual strains. For each population (Figure 1), we sampled one female per isofemale line for a total of 22–24 lines (Table S4). We then evaluated the heterogeneity of the frequencies between the Northern and the Southern populations using a maximum likelihood procedure. Strains are not fully isogenized as evidenced by the heterozygosity of many TEs for presence and absence in many strains (data not shown). We assumed that each tested strain effectively contains two different haploid genomes and that different strains within a tested set come from a panmictic population. The data for each TE in each population come in the form {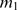, 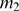, 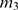} where 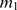 is the number of strains homozygous for the presence of the TE, 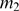 is the number of strains heterozygous for the presence of the TE, and 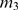 is the number of strains that are homozygous for the absence of the TE. The log-likelihood of observing such data conditional on the frequency *p* is:
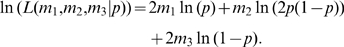
(1)The 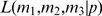 is maximized at the value 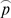:
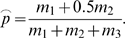
(2)


To determine whether the frequencies in the Northern and Southern populations are different from each other we compare the log-likelihoods of two models. Under H1 we assumed that the frequencies in the two populations are different and estimate them using equation 2 using the data that come from each population separately. We also calculated the two corresponding maximum log-likelihoods. Under H2 we assumed that the frequency of the TE is the same in both populations and estimate this frequency using equation 2 with the combined data from the two populations. We also estimate the maximum log-likelihood under H2. The heterogeneity is detected when the difference between the sums of the two maximum log-likelihood values under H1 and the maximum log-likelihood value under H2 (denoted by ΔL) is greater than 3.84 corresponding to the 5% critical value of the χ^2^ test with one degree of freedom.

### Checking for presence/absence of polymorphic inversions

Three of the four cosmopolitan inversions described in *D. melanogaster* have been characterized at the molecular level. We checked for the presence of these inversions in all the strains analyzed using the following primers: for inversion *In(2L)t* we used the primers described in Andolfatto et al. 1999 [80]. For inversion *In(3R)Payne* we used the primers described in Matzkin et al. 2005 [81]. Finally, for inversion *In(3L)Payne* we used the distal breakpoint sequences described in Wesley and Eanes 1994 [82] to design primers to check for the presence and the absence of the inversion. Primer pair 5′-CCGGATGGACCACATAGAAC-3′ and 5′-CATTCTGGGCCTTATCATCT- 3′ amplifies the standard, but not the inverted chromosome and primer pair 5′-CCGCAAACGAACACTTA-3′ and 5′- GATTATGGACCTAATGAAAGC-3′ amplifies the inverted, but not the standard chromosome.

### Regression analysis

Associations between TE frequencies and latitude were examined using a regression analysis. Only the results of linear regressions are presented because nonlinear patterns were not detected when latitude was treated as a quadratic term. Each of the four Australian populations analyzed (Innisfail, Redland Bay, Coffs Harbour and Melbourne) was treated as a single datapoint. The frequencies of the five analyzed TEs in these four populations are given in Table S3. All frequency data were angular transformed before performing the regression analyses.

Climatic data for weather stations adjacent to collection sites were obtained from the Australian Bureau of Meteorology (www.bom.gov.au; Table S7). The two temperature variables used in the analysis were maximum and minimum temperature. In addition, we also considered rainfall. For all three climatic variables, 20-year averages were used because selection coefficients are small, and frequencies therefore are affected more by long-term climatic patterns than by short term trends [25]. The association between TE frequencies and the different climatic variables was also analyzed using regression.

### Maximum likelihood estimation of selection coefficients

Maximum likelihood estimates for selection coefficients of TE families were derived by comparing observed TE frequencies to those expected under mutation-selection balance, using a simplified version of the approach presented in González et al. 2008 [18]. We model TEs to have codominant fitness: individuals homozygous for a TE have fitness 1+*s* and heterozygotes have fitness 1+*s*/2. The expected population-frequency distribution of a TE family for a panmictic, constant-sized population is then given by
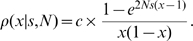
(1)The normalization factor *c* is defined via the condition ∑_x_ρ(x|s,N) = 1, where the sum is taken over the set {1/(2*N*),…,(2*N*−1)/(2*N*)} of possible population frequencies *x* in a diploid population of size *N*.

All TEs in our analysis were originally ascertained in a single sequenced strain [53]. The probability to observe a TE at population-frequency *x* is therefore
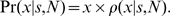
(2)


Our TE frequency data comes in the form *M_i_* = {*m*_1_, *m*_2_, *m*_3_, *m*_4_, *m*_5_, *m*_6_}, where *m*_1_ is the number of North American strain pools where the element is absent, *m*_2_ is the number of pools where it is polymorphic, and *m*_3_ is the number of pools where it is fixed. Counts *m*_4_ and *m*_5_ give the numbers of pools with partial information - those where the element is either absent or polymorphic, and those where the element is either polymorphic or fixed. The numbers of strains vary between 8 and 12 for different pools and in our analysis we adopted an intermediate value of 11 strains per pool, close to the pool average. The error rates of a pool appearing to have the element as either absent or fixed, while it is actually polymorphic, were estimated to be *e*_1_≈0.042 and *e*_2_≈0.010, respectively (Petrov, D.A., Fiston-Lavier, A.-S., Lipatov, M., Lenkov, K. and González, J., unpublished data).

For those pools where we can distinguish perfectly between the three classifications, the probability of *m*_1_ pools classified as absent, *m*_2_ as polymorphic, and *m*_3_ as fixed, is
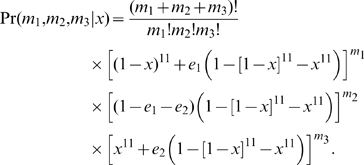
(3)The probability of finding that the element is absent or polymorphic in *m*_4_ pools is
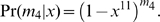
(4)The probability of finding that an element is polymorphic or fixed in *m*_5_ pools is
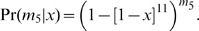
(5)Summing the product of the probabilities (2)–(5) over all possible frequencies *x* yields the probability of a particular observation *M*_i_ for an individual TE,
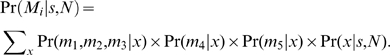
(6)The likelihood-function for the selection coefficient *s* of a TE family is then defined by multiplying the probabilities (6) of all its elements (and thus assuming independence of individual TEs),
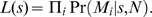
(7)We note that in the regime *N*≫1 and |*s*|≪1, (7) becomes effectively a function of the product *Ns* and we therefore used a fixed value of *N* = 10^4^ for our analysis.

Maximum likelihood estimates and their confidence intervals were obtained numerically by simulated-annealing. The 95% confidence intervals around maximum likelihood estimates *Ns* ^*^ were thereby calculated by solving for *Ns* such that log[*L*(*Ns* ^*^)/*L*(*Ns*)] = 2.512, where we assumed that log-likelihood ratios in our analysis follow a χ^2^ distribution with one degree of freedom.

## Supporting Information

Table S1 Initial dataset.
(0.02 MB XLS)

Table S2 Selection coefficient estimates.
(0.02 MB XLS)

Table S3 Population frequencies of the five TEs analyzed in the four populations collected along the Australian cline.
(0.02 MB XLS)

Table S4 *D. melanogaster* isofemale strains used in this study.
(0.03 MB DOC)

Table S5 Population frequencies of TEs found to be present at low frequencies in Malawi in three additional African populations.
(0.04 MB DOC)

Table S6 Consistency of TE frequencies between years in the two Australian collections analyzed.
(0.02 MB DOC)

Table S7 Climatic data.
(0.01 MB XLS)
