# Supplementary material for: Genome-Wide Patterns of Adaptation to Temperate Environments Associated with Transposable Elements in Drosophila
Source: PLoS Genet. 2010 Apr 8;6(4):e1000905. doi: 10.1371/journal.pgen.1000905 (PMC2851572; doi:10.1371/journal.pgen.1000905)
Supplement: Table S4 — D. melanogaster isofemale strains used in this study. (0.03 MB DOC) [file pgen.1000905.s004.doc]

Table S4. *D. melanogaster* isofemale strains used in this study.

| **Population** | **Strains analyzed** | **Geographical origin** | **Reference** |
| --- | --- | --- | --- |
| Malawi (MW) | 7, 8, 11, 12, 14, 15, 27, 28, 35, 56, 60 | Mwanza, Malawi | David Begun |
| Zimbabwe-1 (ZW-1) | 58, 81, 125, 131, 145, 159, 178, 191, 196 | Lake Kariba, Zimbabwe | Charles Aquadro |
| Zimbabwe-2 (ZW-2) | 104, 109, 122, 140, 141, 142, 144, 149, 155, 156, 177, 183 | Victoria falls, Zimbabwe | Peter Andolfatto |
| Kenya (KY) | 01, 10, 12, 16, 20, 23, 24, 38, 42, 91, 106 | Nairobi, Kenya | Peter Andolfatto |
| Innisfail 2007 (N) | 3, 5, 16, 19, 26, 29, 30, 32, 38, 41, 42, 44, 46, 47, 48, 49, 50, 51, 52, 55, 61, 63 | Queensland, Australia | Ary A Hoffmann |
| Yering Station 2007 (S) | 27, 30, 31, 41, 44, 47, 48, 95, 116, 131, 149, 154, 158, 191, 193, 197, 213, 216, 222, 224, 225, 313 | Victoria, Australia | Ary A Hoffmann |
| Innisfail 2008 (BB) | 2, 4, 9, 10, 15, 17, 18, 19, 24, 25, 27, 29, 36, 37, 38, 42, 43, 44, 46, 47, 49, 52, 108 | Queensland, Australia | Ary A Hoffmann |
| Redland Bay 2008 (MAE) | 1, 2, 3, 4, 5, 6, 7, 9, 11, 12, 15, 16, 17, 18, 19, 24, 27, 29, 34, 37,39,42, 43 | Queensland, Australia | Ary A Hoffmann |
| Coffs Harbour 2008 (CH) | 1, 2, 7, 10, 12, 13, 15, 17, 20, 22, 23, 26, 30, 33, 34, 35, 37, 38, 39, 40, 41, 42, 43 | New South Wales, Australia | Ary A Hoffmann |
| Melbourne 2008 (M) | 1, 7 8, 29, 47, 48, 59, 62, 93, 97, 117, 120, 125, 131, 133, 138, 139, 140, 141,145, 149, 150, 162 | Victoria, Australia | Ary A Hoffmann |
| Rocky Ridge (RR06) | 1, 2, 3, 4, 5, 6, 7, 8, 9, 10, 11, 12, 13, 14, 15, 16, 17, 18, 19, 20, 21, 22, 23, 24 | Bowdoinham, ME, USA | Paul S. Schmidt |
| Watch Me Grow Farms (WMG07) | 101, 102, 103, 104, 105, 106, 107, 108, 109, 110, 111, 112, 113, 114, 115, 116, 117, 118, 119, 120, 121, 122, 123 | Ft. Pierce, FL, USA | Paul S. Schmidt |
