# Supplementary material for: Genome-Wide Patterns of Adaptation to Temperate Environments Associated with Transposable Elements in Drosophila
Source: PLoS Genet. 2010 Apr 8;6(4):e1000905. doi: 10.1371/journal.pgen.1000905 (PMC2851572; doi:10.1371/journal.pgen.1000905)
Supplement: Table S5 — Population frequencies of TEs found to be present at low frequencies in Malawi in three additional African populations. (0.04 MB DOC) [file pgen.1000905.s005.doc]

Table S5. Population frequency of TEs found to be present at low frequencies in Malawi in three additional African populations

| **Flybase ID** | **African pools frequency** | | | **Reference** |
| --- | --- | --- | --- | --- |
|  | **ZW-1** | **ZW-2** | **KY** |  |
| FBti0018880 | 0/10 | 0/10 | 0/10 | González et al. 2008^b^ |
| FBti0019170 | 1/9 | 0/11 | 0/11 | González et al. 2008 |
| FBti0019627 | 0/8 | 0/11 | 0 | González et al. 2008 |
| FBti0019065 | 0/9 | 3/12 | 1/10 | González et al. 2008 |
| FBti0018879 | 0/9 | 0/11 | 0/11 | González et al. 2009^c^ |
| FBti0019079 | 0^a^ | 4/12 | 0 | González et al. 2009 |
| FBti0019133 | 2/8 | 1/11 | 4/11 | González et al. 2009 |
| FBti0019165 | 1/9 | 0 | 4/11 | González et al. 2009 |
| FBti0019604 | 0/7 | 0/10 | 0/8 | González et al. 2009 |
| FBti0020125 | 0 | 2/11 | 0 | González et al. 2009 |
| FBti0020057 | 2/7 | 2/10 | 0 | González et al. 2009 |
| FBti0020056 | 0/8 | 0/9 | 0/10 | González et al. 2009 |
| FBti0020155 | 0 | 1/12 | 1/11 | This work |
| FBti0019771 | 2/11 | 0 | 1/11 | This work |

^a^A “0” entry indicates that only pooled-PCR were performed for that particular TE in that particular pool.

^b^González et al. 2008 [18]

^c^González et al. 2009 [79]
