# Supplementary material for: Genome-Wide Patterns of Adaptation to Temperate Environments Associated with Transposable Elements in Drosophila
Source: PLoS Genet. 2010 Apr 8;6(4):e1000905. doi: 10.1371/journal.pgen.1000905 (PMC2851572; doi:10.1371/journal.pgen.1000905)
Supplement: Table S6 — Consistency of TE frequencies between years in the two Australian collections analyzed. (0.02 MB DOC) [file pgen.1000905.s006.doc]

Table S6. Consistency of TE frequencies between years in the two Australian collections analyzed.

FDR: False Discovery Rate.
